# Effectiveness of standard of care, vs. its combination with reflexology and sham reflexology on preoperative anxiety in patients undergoing elective laparoscopic cholecystectomy: a single-blinded randomized controlled trial

**DOI:** 10.3389/fmed.2025.1634575

**Published:** 2025-09-12

**Authors:** Attias Samuel, Keinan-Boker Lital, Somri Mostafa, Schiff Ariel, Gross Yael, Ben-Arye Eran, Matter Ibrahim, Sroka Gideon, Gavrieli Sagi, Steinberger Dan, Schiff Elad

**Affiliations:** ^1^The Ruth and Bruce Rappaport Faculty of Medicine, Technion, Israel Institute of Technology, Haifa, Israel; ^2^Complementary and Integrative Service, Bnai-Zion Medical Center, Haifa, Israel; ^3^Israel Center for Disease Control, Israel Ministry of Health, Gertner Institute, Sheba Medical Center, Tel Hashomer, Ramat Gan, Israel; ^4^School of Public Health, University of Haifa, Haifa, Israel; ^5^Anesthesiology Department Bnai Zion Medical Center, Haifa, Israel; ^6^Reichman University, Herzliya, Israel; ^7^Integrative Oncology Program, The Oncology Service, Lin, Zebulun, and Carmel Medical Centers, Clalit Health Services, Haifa, Israel; ^8^Surgery Department, Bnai Zion Medical Center, Haifa, Israel; ^9^Internal Medicine B, Bnai Zion Medical Center, Haifa, Israel

**Keywords:** preoperative anxiety, surgery, cholecystectomy, integrative medicine, reflexology

## Abstract

**Background:**

Preoperative anxiety is common. To date, no randomized controlled trial has examined the effectiveness of reflexology in reducing preoperative anxiety in patients undergoing elective laparoscopic cholecystectomy.

**Methods:**

A randomized, single blinded, interventional trial was conducted with 300 patients undergoing elective laparoscopic cholecystectomy, comparing the following three groups: controls receiving standard-of-care (SoC) only (group 1); intervention group receiving reflexology and SoC (group 2); and a group receiving sham reflexology and SoC (group 3). The primary outcome was the mean difference between the three groups in visual analog scale for anxiety (VAS-A). The secondary outcome was a similar analysis confined to patients experiencing baseline moderate-to-high anxiety. In all groups, level of preoperative anxiety was evaluated at entry and exit from the holding room area (HRA). The evaluation was carried out using the VAS-A questionnaire. The study was registered at clinicaltrials.gov (NCT01733771).

**Results:**

101 patients were randomly assigned to the reflexology group, 99 to the sham reflexology arm, and 100 received SoC alone. In all groups, SoC included anxiolytics in 25% of patients received about 2 h before the operation. Baseline anxiety (at entry to HRA) was similar in all groups, averaging 5.3. Between-group analysis comparing the reflexology and sham groups detected 0.8 point difference on 0–10 VAS scale in favor of the reflexology group (*p* = 0.022). Subgroup analysis of patients with moderate to high level of anxiety (VAS-A>4) at baseline (consisting of 75% of study participants), indicated 1.3 points difference (*p* = 0.023).

**Conclusions:**

The study findings suggest that reflexology treatments have a small, yet significant advantage over sham reflexology and over standard-of-care in reducing preoperative anxiety, in patients with moderate-to-high levels of anxiety.

**Clinical trial registration:**

https://clinicaltrials.gov/study/NCT01733771?tab=results, Identifier: NCT01733771.

## 1 Background

Cholecysectomy due to gallstones is one of the most common operations in general surgery (1). Nowadays, this type of surgery is usually done electively in the laparoscopic method laparoscopic cholecystectomy (LC), in Israel and worldwide (2). In recent years, enhanced recovery after surgery (ERAS), an international association of experts in the fields of surgery, anesthesia, and other allied health professions, has led a process of formulating guidelines, based on research evidence, in order to improve peri-operative care (3). This trend reflects an interventionist approach and a global perspective on the public health level in the context of surgery which, among others, highlights the need to address peri-operative symptoms.

The state of preoperative anxiety may affect the course of surgery usage of anesthetics (4), and the recovery period that follows in different aspects, such as pain (5, 6), wound healing (7), nausea and vomiting (8), duration of hospitalization and recovery (9), immune system status (10). Preoperative anxiety is usually evaluated using the visual analog scale for anxiety (VAS-A) questionnaire, which is based on the patient's own reporting and is a reliable, simple and quick method, particularly when the time available for interaction with the subject is limited, as is the case in the holding room area (HRA), a waiting room in which patients stay for 15–50 min before surgery (11–14).

Between 11 and 80% of adult patients undergoing surgery, report experiencing significant (moderate-to-high) preoperative anxiety (15, 16). A controlled, randomized clinical trial on 360 surgical patients showed that 70% of the subjects experienced moderate-to-high levels of anxiety ~1 h before the surgery, despite being treated with the standard-of-care (SoC), which involves administering anxiolytics prior to the surgery at the discretion of the anesthesiologist (17).

Anxiolytics are often administered before surgery, either orally or by intravenous injection. Several studies have proven the efficacy of using anxiolytics as part of premedication before surgery, including midazolam (18), gabapentin (19), diazepam (20) and oxazepam (21). However, data obtained from several other studies cast doubt on their effectiveness (22, 23). Several studies have demonstrated the effectiveness of different complementary medicine treatments, including reflexology, in reducing preoperative anxiety and anxiety before other medical procedures (17, 24, 25).

Reflexology is a touch treatment method which is based on the theory that the entire body is represented in reflex points located on the soles of the feet (or the palms of the hands). A number of studies have examined the impact of reflexology treatments on indices of stress, anxiety and relaxation (17, 26, 27).

A handful of studies compared reflexology treatments with sham reflexology treatments (general foot massage without applying direct pressure to formal reflex points), to counteract the expected placebo effect (28, 29). Such studies are important in order to isolate the treatment's placebo effect and highlight the specific effect, if any, of real reflexology.

Since elective LC is a very common operation, and due to the significance of preoperative anxiety in the context of potential post-operative complications and morbidity, we have examined the effect of reflexology as well as sham reflexology in parallel with SoC treatment which currently involves administration of anxiolytics, on this issue.

## 2 Methods

### 2.1 Type of study and study population

We conducted a single-blinded clinical intervention randomized controlled trial. Trial methods and results were reported according to the 2010 guidelines for consolidated standards of reporting trials (CONSORT) for non-pharmacologic interventions (30).

**Figure 1:**
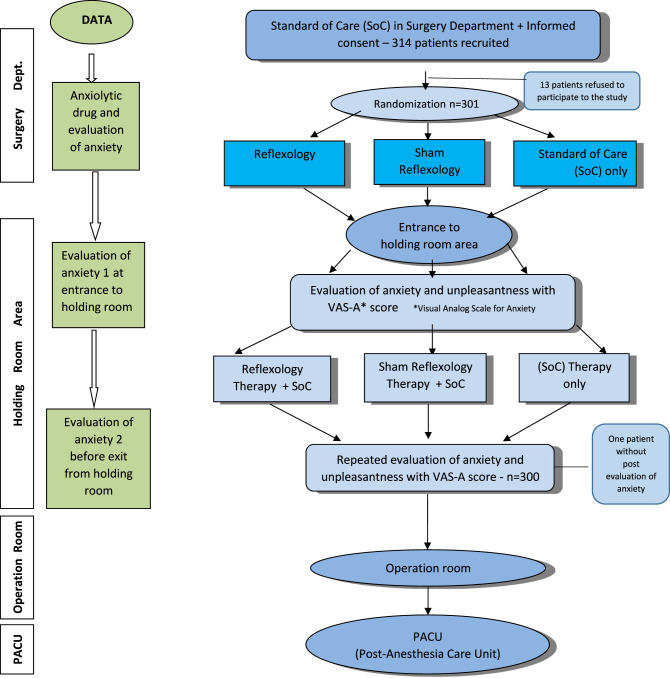
Study procedure.

The trial involved 300 patients awaiting an elective LC, who agreed to participate in the trial and were randomly allocated into three equal groups. Group 1 received the SoC treatment (anxiolytics) at the discretion of the anesthesiologist (*n* = 100). Group 2 received reflexology treatment on top of SoC (*n* = 101), and Group 3 received sham reflexology treatment in addition to SoC (*n* = 99; see Figure 1). The study was single-blinded because while patients in the study arms that involved reflexology did not know whether they were being treated with real reflexology or sham reflexology, and the treatment was described to them as medicinal foot massage rather than reflexology, the therapists, however, did know whether they were giving real or sham reflexology treatment.

Inclusion criteria: (1) patients aged 18 years undergoing LC who consented to the study. Exclusion criteria: (1) patients with a history of obstructive sleep apnea; (2) contraindication for benzodiazepines; (3) hemodynamic instability; (4) patients with feet ulcers; (5) patients undergoing a LC together with another surgical procedure.

### 2.2 Study objective

The primary study objective was to examine the effectiveness of a combined treatment of reflexology on top of SoC in comparison to a combined treatment of sham reflexology on top of SoC and in comparison to SoC treatment alone in reducing preoperative anxiety in elective LC.

### 2.3 Sampling method and study procedure

The study was conducted at the Bnai Zion Medical Center in Haifa. Participants were recruited between July 2016 and October 2019.

Patients that agreed to be included in the study were randomly assigned to one of the three study groups using a designated software (randomization.com; see Figure 1). Patients were asked to rate their level of anxiety and their level of comfort using the visual analog scale (VAS) and visual analog scale for anxiety (VAS-A) questionnaire at the surgery department and in the HRA. Subsequently, patients were told whether they had been assigned to the intervention group involving foot massage—without specifying whether it would be real or sham reflexology—or to the control arm.

### 2.4 Therapeutic approach protocol

The study included three treatment groups:

A. *SoC (group 1)* included premedication about 2 h before the operation with the anxiolytics oxazepam and diazepam, according to the anesthesiology department protocol and at the discretion of the anesthesiologist, regardless of the study arm. It should be clarified that in some cases the anesthesiologist decided not to administer anxiolytics, or the patient chose not to receive them. In cases where premedication was administered, the instructions were to administer 10 mg of diazepam to patients under the age of 65, and 10 mg of oxazepam to patients over 65. This protocol is supported by the study conducted by Pomara et al. (23) which found that older patients taking diazepam tend to suffer from side effects of memory and psychomotor performance impairments.

B. *Reflexology (group 2)* intervention involved a 15-min treatment in the induction room, provided by three reflexologists from the hospital staff. The reflexology protocol was developed through a Delphi method (31). The Delphi expert panel comprised 20 reflexologists with at least 2 years of clinical experience. Using a three-round Delphi process, the first-round open question was developed by four therapists from our Medical Center based on their experience treating preoperative anxiety. Consensus was defined as ≥80% agreement. Topics without consensus were reconsidered in subsequent rounds. Participants were contacted by email, with addresses kept confidential to ensure anonymity and prevent individual dominance (31).

C. *Sham reflexology (group 3)* intervention was provided by two complementary medicine practitioners with knowledge in touch therapy (shiatsu) and included 15 min of gentle, non-specific foot massage. Similarly to the true reflexology protocol, the protocol for this treatment was also determined in a consensus-reaching process among a group of four experienced reflexologists.

### 2.5 Assessment tools

The level of anxiety was evaluated both before and after the treatments, using the VAS-A questionnaire for anxiety and the VAS scale for the level of comfort (rated from 0—not comfortable at all, to 10—very comfortable). VAS Comfort questionnaires were used in order to support the assessment of anxiety by VAS-A. The control group was assessed at the same time points. The questionnaire was given to the patient at the surgery department by a study coordinator. At the HRA questionnaires were given by the nursing staff before and after the intervention, shortly before the patient was transferred to the operating room. Baseline assessment was carried out at the entrance to the HRA through a VAS-A valid anxiety questionnaire for current anxiety level from 0—no anxiety, up to 10—maximum anxiety (14). Similar to pain scale, it is common to divide the anxiety symptoms into categories; low anxiety (VAS-A ≤ 4), moderate to severe anxiety (VAS-A ≥ 4), severe anxiety (VAS-A ≥ 7).

### 2.6 Sample size calculation and statistical analysis

The calculation of the sample size was carried out using GPower software, which examines the sample size with reference to ANOVA variance analysis. The comparison between the three different groups, given a confidence level of 95%, power of 85% and an effect size that is considered medium according to Cohen's 0.25, alongside the definition of one unit of VAS as the low value for clinical significance (32), required a sample size of at least 45 subjects per group for our primary endpoint (mean difference between the three groups in VAS-A). For the secondary endpoint (mean difference between the three groups for patients with VAS above four), and for stratification of age, sex, as well as according to the administration of anxiolytics, ~100 subjects were recruited to each group. Since the result distribution was not normal, a variance test was conducted using Kruskal–Wallis' non-parametric test to compare the average change in preoperative anxiety in the induction room. Due to multiple comparisons (between three groups), the *p*-value in these tests was 0.014 in order to overcome the false discovery rate (FDR), and *p* < 0.05 for the purpose of bilateral comparison (33). Of note, both per-protocol and intention-to-treat analyses were planned, but since there was no patient cross-over, there was no need to differentiate between the analyses.

### 2.7 IRB approval

The study was approved by the local IRB (Helsinki Committee), approval no. BNZ-0041-09 and is also registered at clinicaltrials.gov (NCT01733771).

## 3 Results

We approached 314 eligible candidates. A total of 300 patients were included in the study. The rate of consent to participate in the study was very high−95.5% (300/314). Thirteen patients (4.1%) refused to participate in the study, and one patient (0.3%) could not undergo an evaluation of his anxiety level after the treatment because he was taken to the operating room before being asked again.

### 3.1 Patients' characteristics

The characteristics of the patients in the entire study population are presented in Table 1. No statistically significant differences were found between the different groups. All patients were able to receive the anxiolytics oxazepam and diazepam.

**Table 1:** Study population characteristics.

**Parameter**	**Total no. of patients**	**R**	**SR**	**SoC**	***p*-Value**
	***N*** = **300**	***n*** = **101**	***n*** = **99**	***n*** = **100**	
	**Mean (SD)**	**Mean (SD)**	**Mean (SD)**	**Mean (SD)**	
Age, years (average ± SD)	49.8 ± 15.6	51.2 ± 14.5	49.9 ± 16.9	48.5 ± 15.3	*p* = 0.46
BMI	28.4 ± 5.4	28.3 ± 5.9	28.6 ± 5.3	28.3 ± 5.1	*p* = 0.93
Sex (female)	188 (63%)	60 (59%)	62 (63%)	66 (66%)	*p* = 0.63
Surgery in the past	187 (64%)	69 (70%)	54 (55%)	64 (67%)	*p* = 0.07
An inflammatory process that required hospitalization before surgery	127 (46%)	49 (50%)	43 (47%)	35 (41%)	*p* = 0.4
Patients who received anxiolytics (diazepam or oxazepam)	72 (25%)	23 (23%)	24 (25%)	25 (26%)	*p* = 0.86
**Comorbidity**					
Cardiovasculary	103 (35%)	37 (37%)	32 (33%)	34 (34%)	*p* = 0.86
Gastroenterology/hepatobiliary	38 (13%)	17 (17%)	10 (10%)	11 (11%)	*p* = 0.32
Renal/urology	23 (8%)	6 (6%)	10 (10%)	7 (7%)	*p* = 0.65
Metabolic/endocrinology	152 (51%)	53 (53%)	48 (49%)	51 (51%)	*p* = 0.92
Hemato-oncology	8 (18.2%)	5 (11.4%)	2 (4.5%)	2 (4.9%)	*p* = 0.37
Neurology	3 (1%)	3 (3%)	–	–	–
Others	53 (18%)	15 (15%)	20 (20%)	18 (18%)	*p* = 0.63
Baseline VAS-A in surgery department mean (SD)	4.2 ± 2.6	4.2 ± 2.6	4.4 ± 2.6	3.9 ± 2.6	*p* = 0.33

p = 0.014 (between three groups). R, reflexology; RS, sham reflexology; SoC, standard of care.

### 3.2 Baseline anxiety and comfort levels in the ward and HRA

The VAS-A anxiety evaluation performed in the ward revealed an average anxiety level of 4.2 in the entire sample. Upon entering the HRA ~2–6 h later, an average anxiety level of 5.3 was measured, an increase of 1.1 points in the VAS-A scale. The average VAS comfort level, the complementary index to the VAS-A, was 5.1 upon entering the HRA. As part of the data processing, we focused on the group of patients whose anxiety level was rated as moderate-high when they arrived at the HRA, prior to the intervention (a VAS-A score higher than 4). This group included a total of 221 patients (74%). Among these patients, the average level of anxiety upon entering the HRA and before the intervention was 6.5 (Table 2).

**Table 2:** Baseline outcomes evaluation in holding room area per groups and per categories of VAS-A and VAS comfort.

**VAS comfort and VAS-anxiety baseline in holding room area**	***N***	**Mean (SD)**	***p*-Value between 3 groups**
VAS comfort (0–10)	300	5.1 (2.2)	*p* = 0.71
R	101	5.2 (2.3)	
SR	99	5.1 (2.2)	
SoC	100	5.1 (2.2)	
VAS-A (0–10)	300	5.3 (2.7)	*p* = 0.47
R	101	5.1 (3.2)	
SR	99	5.5 (2.4)	
SoC	100	5.1 (2.7)	
VAS-A ≥4 moderate to severe anxiety	212	6.47 (1.9)	*p* = 0.19
R	61	6.8 (1.9)	
SR	83	6.2 (1.8)	
SoC	68	6.5 (1.9)	
VAS-A ≥7 severe anxiety	73	8.93 (1.0)	*p* = 0.61
R	25	9.08 (0.9)	
SR	23	8.85 (1.0)	
SoC	25	8.84 (1.0)	
VAS-A < 4 low anxiety	78	1.71 (0.97)	*p* = 0.57
R	33	1.57 (0.94)	
SR	16	1.88 (1.08)	
SoC	29	1.76 (0.95)	

R, reflexology; SR, sham reflexology; SoC, standard of care. p = 0.014 (between three groups).

With regard to the correlation between the VAS-A anxiety score and the VAS comfort score measured in the “Before” evaluation at the HRA in the entire study population, the Pearson coefficient shows a strong negative correlation between anxiety and comfort: the higher the levels of anxiety, the lower the levels of comfort. The strength of the correlation is *r* = −0.714, *p* < 0.0001.

### 3.3 Pre-and post-intervention anxiety assessment

Table 3 shows the change in the average level of anxiety “Before” and “After” the intervention in the HRA, for each type of treatment. With the complementary medicine treatments, the average differences in anxiety level are negative, meaning that a [statistically significant] decrease was observed in the level of anxiety between the evaluation “Before” and the evaluation “After” the intervention. With the SoC alone, the difference is positive, indicating that a certain increase in anxiety was observed in the second evaluation.

**Table 3:** Mean difference anxiety in holding room area by groups and by level of initial anxiety level.

**Mean difference VAS-A**	***N***	**Mean difference (SD)**	**Median**	***p*-Value between three group**	***p*-Value between two group**	**95% CI**
**VAS-A all anxiety levels**						
R	101	−2.83 (2.12)	2.25	*p* < 0.0001	SoC vs. SR *p* < 0.0001	[(−3.25)–(−2.41)]
SR	99	−1.99 (1.85)	2.00		SoC vs. R *p* < 0.0001	[(−2.36)–(−1.62)]
SoC	100	0.24 (1.06)	0.00		SR vs. R *p* = 0.021	(0.03–0.46)
**VAS-A** ≥**4 moderate to severe anxiety**						
R	66	−3.58 (2.14)	3.00	*p* < 0.0001	SoC vs. SR *p* < 0.0001	[(−4.11)–(−3.06)]
SR	81	−2.16 (1.93)	2.00		SoC vs. R *p* < 0.0001	[(−2.58)–(−1.73)]
SoC	70	0.20 (1.3)	0.00		SR vs. R *p* = 0.022	[(−0.07)–(0.47)]
**VAS-A** ≥**7 severe anxiety**						
R	25	−4.84 (2.49)	5.00	*p* < 0.0001	SoC vs. SR *p* < 0.0001	[(−5.87)–(−3.81)]
SR	22	−3.27 (2.02)	3.50		SoC vs. R *p* < 0.0001	[(−2.38)–(−4.17)]
SoC	25	0.20 (0.70)	0.00		SR vs. R *p* = 0.35	[(−0.09)–(0.49)]
**VAS-A**<**4 low anxiety**						
R	32	−1.28 (0.86)	1	*p* < 0.0001	SoC vs. SR *p* < 0.0001	[(−1.59)–(−0.97)]
SR	16	−1.12 (1.14)	1		SoC vs. R *p* < 0.0001	[(−1.73)–(−0.51)]
SoC	29	0.34 (0.89)	0.00		SR vs. R *p* = 1.00	(0.004–0.68)

R, reflexology; SR, sham reflexology; SoC, standard of care. p = 0.014 (between three groups). p < 0.05 (between two group).

**Figure 2:**
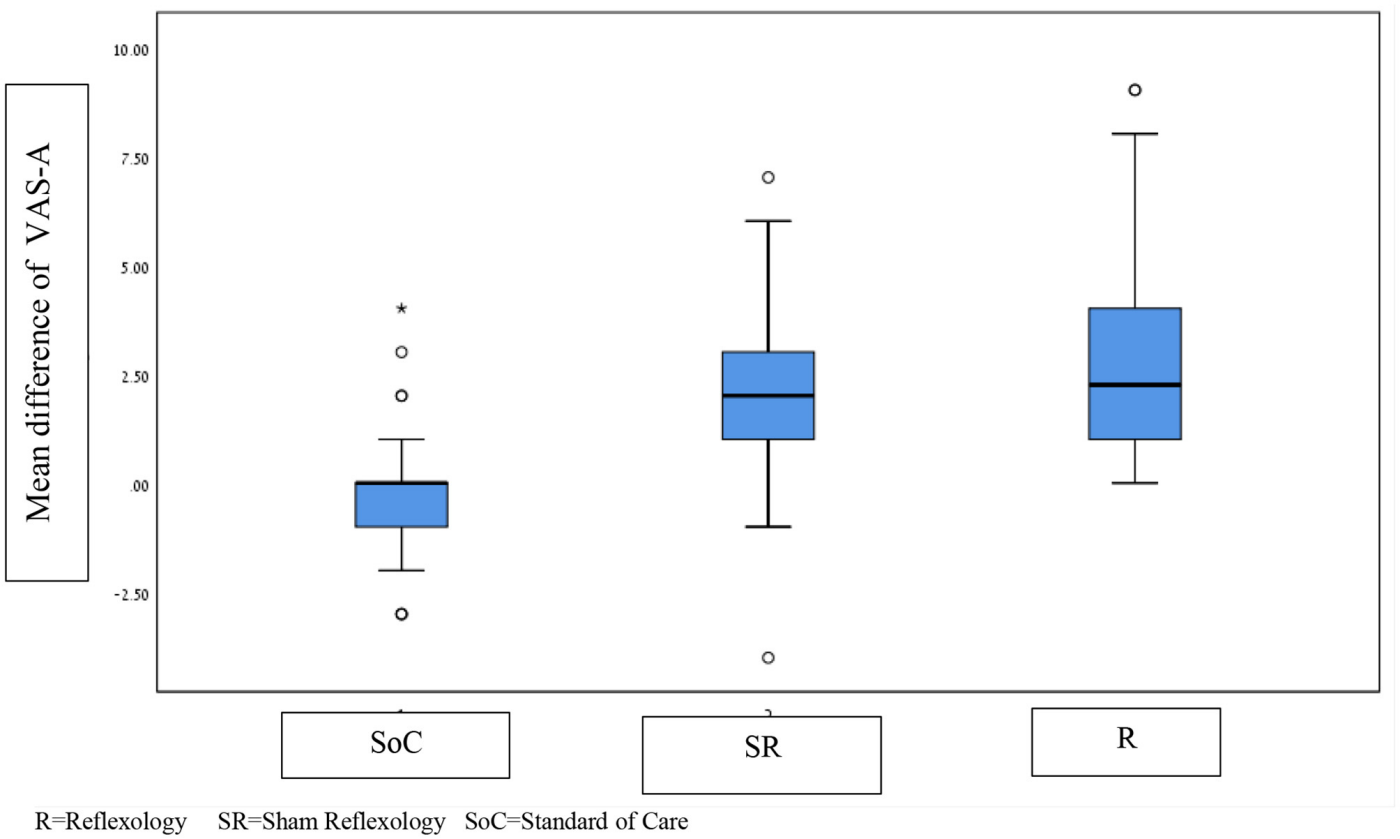
Differences in the average anxiety scores in the holding room area in the three groups. R, reflexology; SR, sham reflexology; SoC, standard of care. *Meaning that several patients had changes in VAS-A far outside the main distribution.

Bilateral comparisons show a statistically significant difference in the reduction of anxiety levels after intervention between the reflexology and sham reflexology treatments (0.8) *p* = 0.022, a finding that is significant but has no clinical significance. A statistically significant difference was also observed in the comparisons between the SoC group and the sham reflexology group (2.2) and between SoC and reflexology groups, *p* < 0.0001, with clinical significance. Figure 2 describes each of the treatments using a box-plot and the Kruskal–Wallis test.

### 3.4 The change in average anxiety level in the HRA among patients with initial moderate-to-high anxiety level VAS-A ≥4

**Figure 3:**
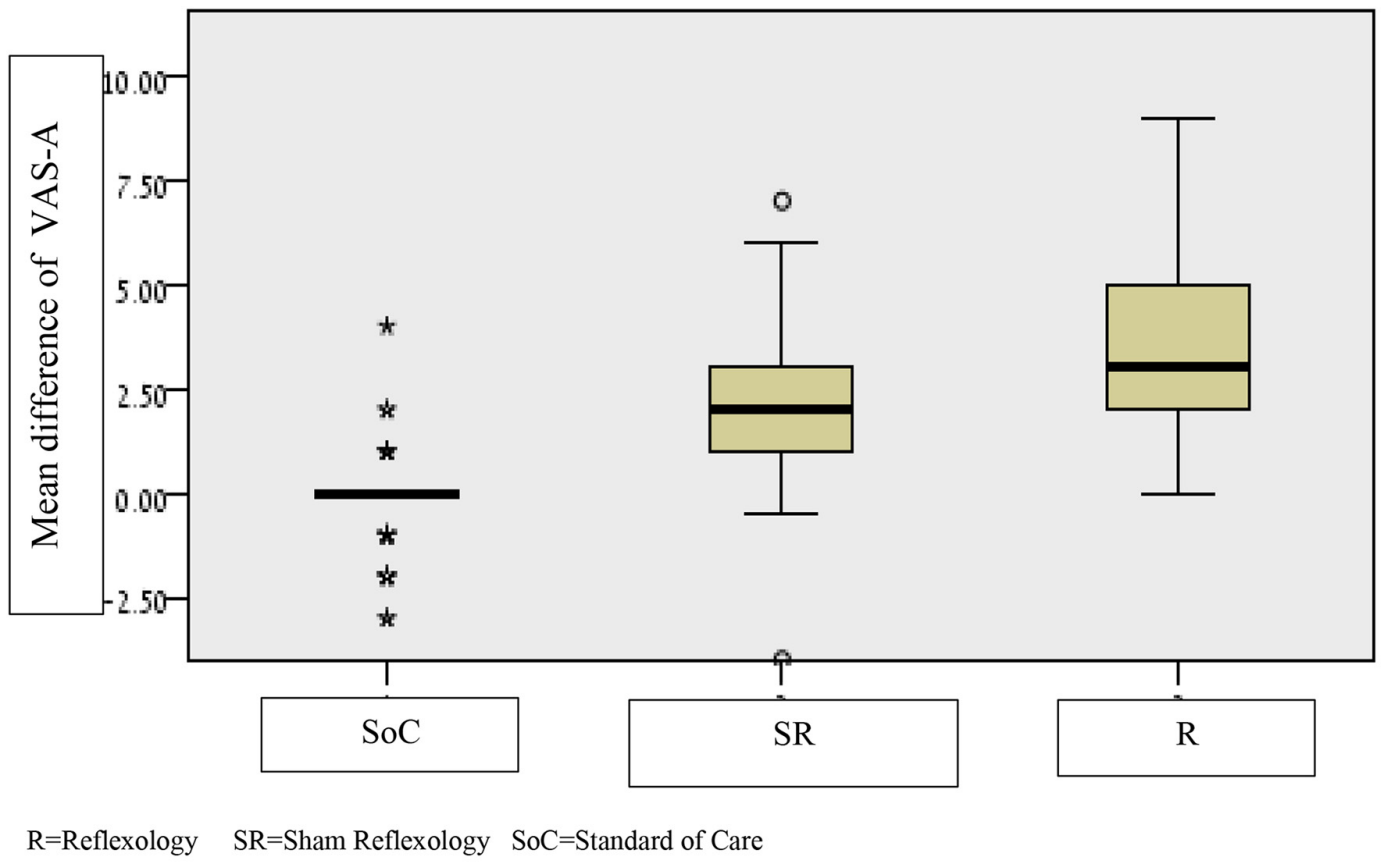
Differences in average anxiety scores before and after the holding room area intervention in the three groups, restricted to patients with moderate-high anxiety VAS-A ≥4. R, reflexology; SR, sham reflexology; SoC, standard of care. *Meaning that several patients had changes in VAS-A far outside the main distribution.

Once again, we conducted a Kruskal–Wallis test between the study groups for participants with VAS-A ≥4, while looking at the difference in anxiety levels between “Before” and “After” the intervention as an outcome. A statistically significant difference emerged between reflexology and sham reflexology (1.3) *p* = 0.022, with a clinical significance defined as minimal to small. Moreover, a significant difference was found between the SoC group and the sham reflexology group, and between SoC and reflexology groups, *p* < 0.001 (see Table 3 and Figure 3).

### 3.5 Data analysis of the study population stratified by use of anxiolytics

Twenty-five percent of the study population received anxiolytics about 2 h before the operation. No significant differences were found between the different study arms with regard to the administration of anxiolytics (see Table 1). Analysis of the effect of anxiolytics will be published in a separate article.

### 3.6 Safety issues

No abnormal reactions to the reflexology or sham reflexology were reported during or immediately after the treatments. In four cases, mild involuntary reactions of foot twitches were observed during the reflexology treatment.

## 4 Discussion

The aim of this study was to examine the effect of reflexology as well as sham reflexology in parallel with SoC treatment on pre-operative anxiety in patients undergoing elective laparoscopic cholecystectomy. The study findings indicate that reflexology treatments have a clinically modest advantage over sham reflexology in reducing preoperative anxiety in patients with a moderate-to-high baseline level of anxiety (VAS-A ≥4), and that both interventions have a considerable advantage over the standard of care, regardless of anxiolytics administration.

To the best of our knowledge, this study is the first to evaluate anxiety levels among cholecystectomy patients pre- and post-reflexology intervention immediately before entering the operating room thus, it reliably indicates anxiety levels directly before the operation, following administration of SoC.

### 4.1 The findings in light of previous studies

In the present study, the initial average anxiety levels measured in the HRA using the VAS-A scale before entering the operating room and before the intervention were clinically significant (5.3), were validated by the complementary VAS-Comfort scale and were similar to the average measured in studies conducted by Perks (34) and Mackenzie (35). The average level of anxiety measured in the present study is also similar to the results of a study conducted using a similar outline, which evaluated the level of anxiety in the HRA—showing an average VAS-A score of 5.5 in 360 patients undergoing different types of operations (17). The breakdown of anxiety severity was also similar to previous studies, with 70% of the study population exhibiting initial moderate-to-high anxiety levels (VAS-A score of 4–10), which require special attention from the medical staff due to the risk of peri-operative complications.

The clinical benefit observed with reflexology, as compared to sham reflexology and SoC, is a finding unique to our study. An extensive literature review has shown that most of the studies that examined the efficacy of non-pharmacological treatments for preoperative anxiety did not include a sham intervention arm (17, 35, 36). Out of three studies that did include a sham reflexology arm, all with relatively small sample sizes (15–73), only one (29) reported that real reflexology had a relative advantage over sham reflexology (28, 37).

### 4.2 Timing the treatment of preoperative anxiety

Our findings show an average increase of one VAS-A unit from the moment the elective-surgery patients are admitted to the surgery department until they are taken to the HRA, where the level of anxiety reaches its peak. Therefore, it appears appropriate to treat preoperative anxiety from the moment patients are informed of the surgical intervention at the preoperative clinic. At this stage, surgeons play a crucial role in the way they explain the procedure, including its benefits and potential risks (38). Approximately 2 weeks before surgery, patients typically attend a preoperative clinic, where it is appropriate to consider pharmacological, psychological, and/or complementary interventions, particularly for those with high anxiety. Finally, during hospitalization prior to surgery, proactive interventions are recommended both in the department and in the HRA. Future studies should evaluate the optimal timing and modalities for such interventions.

### 4.3 Reflexology and sham reflexology

The study demonstrated a clear therapeutic advantage of both true and sham reflexology over the standard of care in reducing preoperative anxiety. Reflexology also showed a relatively small but significant advantage over sham reflexology in patients with moderate-to-high baseline anxiety. These findings highlight the potential benefits associated with touch-based treatments. They also indicate that the Delphi process, used to standardize this intervention, was appropriate. This further justifies its use in the absence of formal practice guidelines. It is important to note that, although statistically significant, the advantage of true reflexology over sham reflexology is clinically modest on the VAS scale. Nevertheless, the large sample size supports the robustness of this finding. Future studies should aim to identify patient subgroups that may derive greater benefit from true reflexology. In addition, *foot massage* is defined as a non-specific massage of the feet; however, reflex points used in reflexology may sometimes be stimulated unintentionally. In contrast, the sham reflexology protocol in our study was carefully designed to avoid applying pressure to reflex points associated with relaxation. We reviewed the literature to evaluate the impact of foot massage, which may resemble our sham protocol, and found no studies assessing its efficacy for preoperative anxiety. The few studies that examined the effect of foot massage on preoperative anxiety involved complex interventions, such as patient education, preventing isolation of the effect of foot massage (39).

### 4.4 Study limitations and strengths

This study has some limitations that need to be considered. First, it was performed in one medical center. However, the department in which the study was carried out performs a considerable number of LC in the northern part of Israel. In addition, the therapists participating in our study knew that they were giving a sham treatment. Although not assessed in this study, this could have had an unconscious effect on the therapist and on his/her confidence in the treatment, and the patients might have sensed it. Furthermore, although anxiety is a multifaceted experience, we only used the VAS-A tool for its assessment in our study, since it is applicable and practical in the setting we explored. Future studies should incorporate questionnaires, which assess complex elements of the anxiety experience. Objective data related to physiological expressions of anxiety, such as heart rate variability, blood cortisol levels, and other indicators, would have supported our findings. However, we included in the questionnaire an aspect of the patient's comfort in order to assess the reliability of the reported level of anxiety and the correlation between the two variable was high.

Having said that, the study has some significant strengths that should be mentioned. It had a controlled, randomized design, which reduces the potential for selection and information biases and for confounding. Importantly, we have included a study arm focusing on sham reflexology, which neutralized the non-specific placebo effect of the real treatment that has been investigated. In addition, the department in which the study was carried out has a patient population characterized by socio-demographic diversity, which increases the generalizability of our findings. Furthermore, the high response rate (over 95%) significantly reduces the potential for a volunteer bias.

## 5 Conclusion

Our randomized controlled study indicates that reflexology in combination with standard of care reduces preoperative anxiety in patients undergoing elective LC. The modest relative advantage of reflexology over sham reflexology was particularly evident in patients experiencing moderate-to-high baseline levels of anxiety (VAS-A≥4). Future studies should evaluate point specificity, best timing and manipulation, as well as mechanism of action in order to identify the specific effects of reflexology.

In addition, we have found out that both reflexology (+SoC) and sham reflexology (+SoC) were superior to SoC alone in reducing pre-operative anxiety, suggesting that guidelines regarding the current SoC protocols may need rethinking.

## Data Availability

The raw data supporting the conclusions of this article will be made available by the authors, without undue reservation.
